# The Generated Entropy Monitored by Pyroelectric Sensors

**DOI:** 10.3390/s18103320

**Published:** 2018-10-03

**Authors:** Chun-Ching Hsiao, Bo-Hao Liang

**Affiliations:** 1 Department of Mechanical Design Engineering, National Formosa University, No. 64, Wunhua Rd., Huwei Township, Yunlin County 632, Taiwan; a0910690097@gmail.com; 2 Smart Machine and Intelligent Manufacturing Research Center, National Formosa University, No. 64, Wunhua Rd., Huwei Township, Yunlin County 632, Taiwan

**Keywords:** entropy, pyroelectric effect, sensor, energy conversion, failure

## Abstract

Entropy generation in irreversible processes is a critical issue that affects the failure and aging of electrical, chemical or mechanical systems. The promotion of energy conversion efficiency needs to reduce energy losses, namely to decrease entropy generation. A pyroelectric type of entropy detector is proposed to monitor energy conversion processes in real time. The entropy generation rate can be derived from the induced pyroelectric current, temperature, thermal capacity, pyroelectric coefficient and electrode area. It is profitable to design entropy detectors to maintain a small thermal capacity while pyroelectric sensors minimize geometrical dimensions. Moreover, decreasing the electrode area of the PZT cells could avoid affecting the entropy variation of the measured objects, but the thickness of the cells has to be greatly reduced to promote the temperature variation rate and strengthen the electrical signals. A commercial capacitor with a capacitance of 47 μF and a maximum endured voltage of 4 V were used to estimate the entropy to act as an indicator of the capacitors’ time-to-failure. The threshold time was evaluated by using the entropy generation rates at about 7.5 s, 11.25 s, 20 s and 30 s for the applied voltages of 40 V, 35 V, 30 V and 25 V respectively, while using a PZT cell with dimensions of 3 mm square and a thickness of 200 μm.

## 1. Introduction

Output power is to be maximized in energy generation processes. The loss of low-grade thermal energy is maximized during energy conversion. The energy conversion efficiency of electrical motors, combustion engines, refrigerators varies. Electrical motors can reach up to 60% efficiency. These systems usually undergo processes of chemical reactions, aging and failure. Entropy generation can be directly assessed to monitor the failure and aging of working devices, systems, apparatus and furnaces. Energy loss decreases the efficiency and concerns entropy generation. In other words, entropy generation indicates an energy loss in chemical reactions and energy conversions, further representing thermodynamic efficiency. Moreover, the maximum efficiency for energy conversion in a chemical reaction is the ratio of the change in the Gibb’s free energy and the change in enthalpy. The Gibb’s free energy includes a term for the heat of the reaction and a term for the heat value of the entropy change. Hence, the chemical efficiency is lower for a greater entropy change and a higher temperature [1,2,3,4,5]. Moreover, the relationship between entropy generation rate and degradation of solids has been proven experimentally and mathematically [6,7,8,9,10]. Furthermore, entropy generation is monitored in real time and that can be interesting for applications ranging from energy conversion management to system aging prediction for further estimating the failure and aging of electrical or mechanical systems.

Pyroelectricity is the property of a material wherein a temperature change causes a change in electrical polarization. In other words, the pyroelectric effect converts time-dependent temperature fluctuations into electricity. Pyroelectric devices have been widely commercialized as temperature sensors, fire detectors, infrared sensors, pollution monitors, hot image detectors, intruder alarms and for gas analysis. The generation of free charges on the surface of pyroelectric cells perpendicular to the direction of polarization is induced by temperature variations applied to pyroelectric materials. A pyroelectric current is generated by connecting the surfaces of the cells via a conducting medium. Polarization decreases due to re-orientation of the dipole moment when the pyroelectric materials are heated (d*T*/d*t* > 0). This thus generates an electrical current in an external circuit. On the contrary, polarization increases as the dipoles become oriented when the pyroelectric materials are cooled (d*T*/d*t* < 0). This causes a current flow in the reverse direction. At a steady state (d*T*/d*t* = 0), the polarization is constant, and no current is generated. Furthermore, much literature has been dedicated to the study of pyroelectric-based energy harvesting [11,12,13,14,15,16]. A pyroelectric cell can simultaneously generate a larger induced voltage and greater current generation, which is profitable to enhance the efficiency of pyroelectric harvesters. Siao et al. [11] used a lead zirconate titanate (PZT) strip cell fabricated by a precision dicing saw to enhance the efficiency of pyroelectric harvesters. The strip pyroelectric cell with a high-narrow cross section was able to greatly absorb thermal energy via the side walls of the strips, thereby inducing lateral temperature gradients and increasing the temperature variation rates in a thicker pyroelectric cell. However, a larger electrode area of about 50% was lost in the strip’s structure, further decreasing the induced current and charge. Hsiao et al. [12] used a low-cost sandblast etching apparatus to fabricate a high aspect ratio micro-pattern in a thicker bulk PZT pyroelectric cell to further improve the heat transfer and pyroelectric energy transformation by adopting a lower equivalent capacitance to enhance the induced voltage, while introducing lateral temperature gradients to promote the temperature variation rate, the induced charge and current.

The induced current of the pyroelectric cells is based on the pyroelectric effect, which converts temporal temperature variations to corresponding electrical outputs. The pyroelectric current (*I_p_*) and charge (*Q_p_*) are given by [17]:

*I_p_* = d*Q_p_*_/_d*t* = *η* × *p* × *A* × d*T*/d*t* (1)

where *η* is the absorption coefficient of radiation; *A* is the electrode area; d*T*/d*t* is the temperature variation rate of the pyroelectric material and *p* is the pyroelectric coefficient of the pyroelectric material. Integrating the pyroelectric current over time, the induced charge (*Q_p_*) can be inferred as:

*Q_p_* = ∫ *I_p_* d*t* = *η* × *p* × *A* × Δ*T* (2)

Δ*T = T_f_ − T_i_* shows the temperature evolution; *T_f_* and *T_i_* represent the temperatures at the final (*f*) and initial (*i*) times. A thicker pyroelectric material tends to generate a higher induced voltage due to a lower pyroelectric capacitance; the voltage possesses a gentler generation rate due to a larger thermal capacity. Oppositely, a thinner pyroelectric material tends to generate a lower induced voltage due to a larger equivalent capacitance, while the induced current possesses a steeper generation rate due to a smaller thermal capacity.

For the determination of entropy (*S*), heat (*Q*) and temperature (*T*) need to be measured simultaneously. The change in *S* can be expressed as the heat exchanged (*δQ*) in the material at a certain temperature (*T*):

d*S* = *δQ/T* (3)

The relationship between heat and temperature in pyroelectric materials can be expressed as:

*C_p_* = d*Q*/d*T* (4)

where *C_p_* is the thermal capacity. The induced pyroelectric current can be rewritten as:

*I_p_* = *η* × *p* × *A* × d*T*/d*t* = *η* × *p* × *A* × (d*Q*/d*t*)/*C_p_* (5)

Therefore, the entropy generation rate in the pyroelectric cell (*Ś_p_*) at temperature (*T*) can be expressed as:

*Ś_p_* = d*S*/d*t* = *C_p_* × *I_p_*/(*η* × *p* × *A × T*) (6)

The entropy variation (Δ*S*) can be obtained by integrating the induced pyroelectric current over time:

Δ*S* = ∫ (d*S*/d*t*) d*t* = ∫ {*C_p_* × *I_p_*/(*η* × *p* × *A × T*)} d*t* = ∫ *C_p_* d*T/T* = *C_p_* ln (*T_f/_T_i_*) (7)

The temperature of pyroelectric cells varies in the process. Substituting (2) into (7) can be rewritten as the following:

Δ*S* = ∫ {*C_p_* × *I_p_*/(*η* × *p* × *A*)/(*T_i_* + ∫ (*I_p/_*(*η* × *p* × *A*)) d*t*)} d*t* (8)

Hence, both the entropy generation rate (d*S*/d*t*) and the variation (Δ*S*) can be inferred from the pyroelectric devices [18]. Moreover, Cuadras et al. [19] designed a circuit to evaluate the entropy generation of the capacitors from electric systems. The entropy generation rate of the capacitors (*Ś_e_*) from electric systems can be written as [20]:

*Ś_e_* = *P_e_/T* = *I_e_* × *V_e_/T* (9)

where *P_e_* is the total input electrical power, and *I_e_* and *V_e_* are the current and voltage drop between the external terminals, respectively. In the present study, we attempted to use pyroelectric devices to measure the entropy rate and variation by monitoring the temperature fluctuations, the induced pyroelectric current and the heat in the process from the thermal capacity. Although it is profitable to design entropy detectors by minimizing the geometrical dimensions of the pyroelectric cells to maintain a small thermal capacity for further improving thermal conduction, the electrical signals of undersized cells can hardly be measured because they increase the inaccuracy in the entropy estimation. Pyroelectric cells with various geometries were adopted for discussion on the evaluation of the entropy rate and variation. Pyroelectric cells acting as entropy sensors were further applied to commercial capacitors for estimating the entropy generation and predicting the time-to-failure in the capacitors. Comparing *Ś_p_* by the pyroelectric cells with *Ś_e_* by the electric systems could validate the pyroelectric-type entropy sensors.

## 2. Materials and Methods

**Figure 1:**
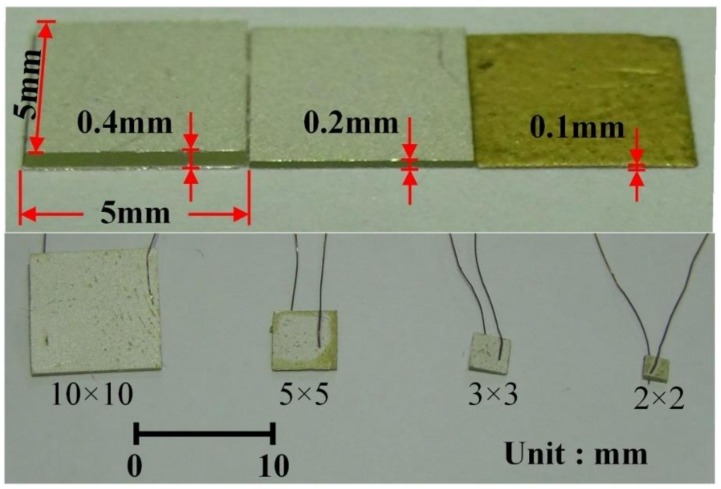
The fabricated PZT cells with various geometries.

The pyroelectric cell used in the present study was a freestanding bulk PZT material. Commercial PZT sheets, with dimensions of 45 mm (length) × 45 mm (width) × 0.4 mm (thickness) and 45 mm × 45 mm × 0.2 mm, were provided by ELECERAM TECHNOLOGY Co., Ltd. (Taoyuan, Taiwan). The pyroelectric cell was a sandwich frame, while the PZT layer was sandwiched in between the top and the bottom electrodes. A 7 μm thick silver layer was adopted to form both electrodes. The properties of the bulk PZT sheet are described in Table 1. A dicing saw (DS-150 II, EVERPRECISION TECH Co., Ltd., New Taipei, Taiwan) was used to slice the PZT sheets into four sizes: 2, 3, 5 and 10 mm squares. Sandblast etching has been used to fabricate various geometries and thicknesses of PZT pyroelectric cells [12,21,22]. A sandblast etching machine (Shang-Po Sander Co. Ltd., New Taipei City, Taiwan) was adopted to process PZT sheets with various thicknesses, as shown in Figure 1.

The heat source entropy generation was used to inspect the pyroelectric-type entropy sensors with various geometries. The heat source is created by using silicone oil heated to about 70 °C. A temperature-controlled ceramic hotplate (IKA C-MAG HS 7, Königswinter, Germany) was adopted to heat a beaker filled with 150 mL silicone oil. The beaker was insulated by a ceramic jacket to reduce thermal loss to the surrounding environment. Silicone oil is an excellent electrical insulator and non-flammable, so it does not affect the electrical signals of the PZT cells. The most important component in silicone oil is polydimethylsiloxane which possesses high thermal stability and lubricating properties. Moreover, silicone oil is widely used in laboratories for heating baths (oil baths) placed on top of hotplate stirrers due to its temperature stability and good heat-transfer characteristics. A PZT entropy sensor was placed in the silicone oil to measure the volume entropy. Heat is transferred to the PZT cells from every direction. The PZT cells were initially allowed to lie at room temperature, then, they were dipped into the thermal bath of silicone oil.

**Figure 2:**
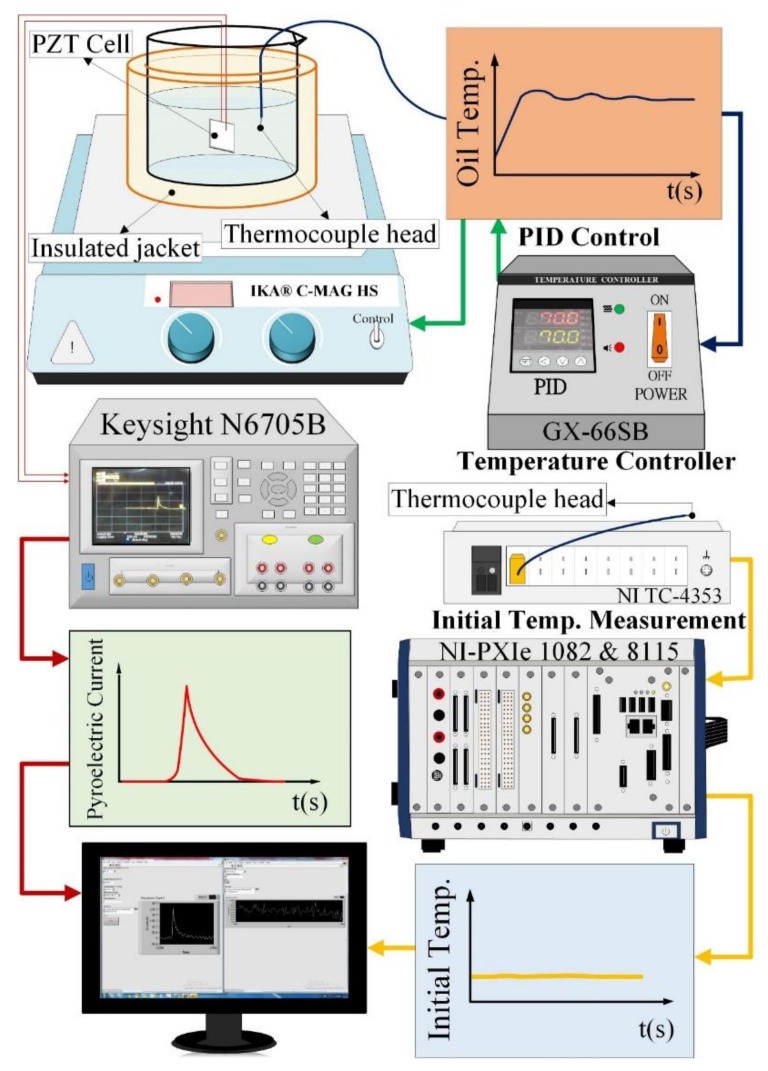
A measurement setup for estimating the PZT entropy sensors.

A pyroelectric device is usually considered a source of current parallel to its equivalent capacitance and resistance. The induced current, voltage and temperature of the PZT cells were simultaneously acquired and recorded by a precise measurement apparatus (Keysight N6705B, Santa Clara, CA, USA) and a NI LabVIEW system (NI-PXIe 1082 and 8115). The induced charge of the PZT cells was calculated by integrating the induced pyroelectric current over time via a numerical integration of Simpson’s rule. Moreover, the temperature in the silicone oil and air was measured using Type K (Chromel/Alumel) thermocouple sensors. A NI PXIe-4353 temperature input module was used to connect the thermocouple sensors with the NI-PXIe 1082. The temperature sensors were also used to settle to the initial temperature of the pyroelectric cells. Furthermore, the temperature evolutions of the PZT cells were estimated by the induced current profile of the cells via (2). The inner temperature of the PZT cell could only be appraised by integrating the induced current over time. An integral measurement coupled thermal and electrical system is depicted in Figure 2.

**Figure 3:**
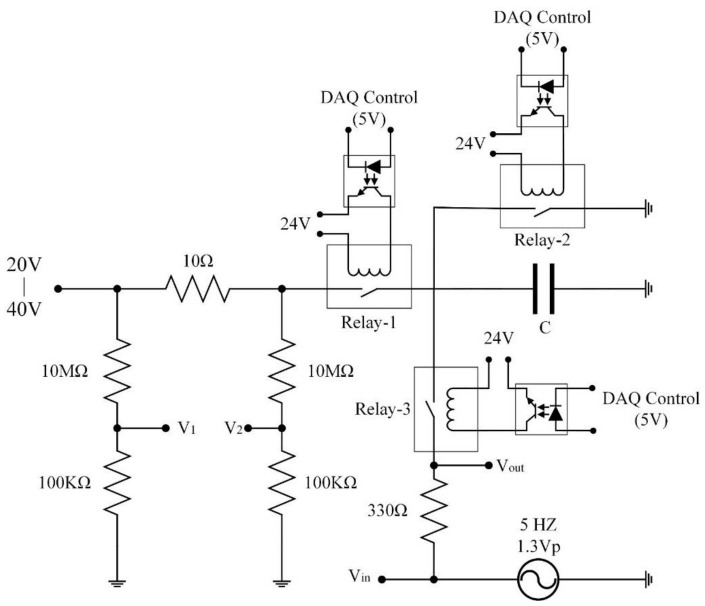
An electrical system used to evaluate the entropy generation in commercial capacitors.

**Figure 4:**
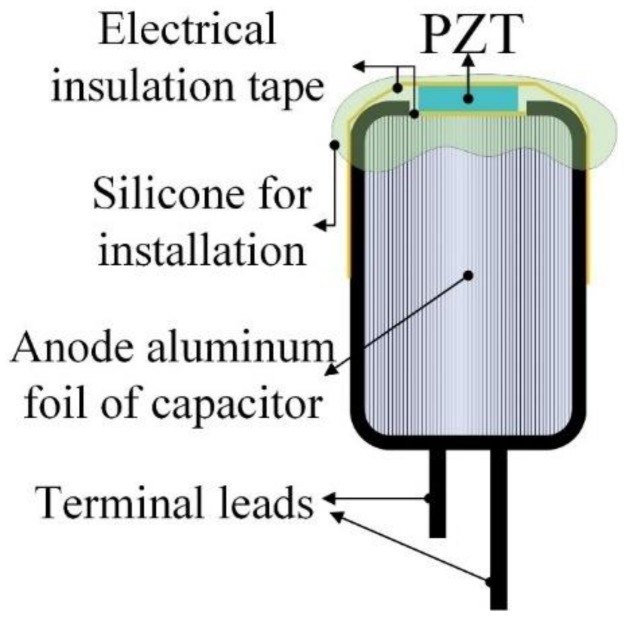
Schematic diagram for the PZT cell installed on the top of commercial capacitors.

A PZT entropy sensor was also used to estimate the lifetime and failure modes of commercial electrolytic capacitors. The capacitor possessed a capacitance of 47 μF and a maximum endured voltage of 4 V. A circuit [19] was designed to appraise the power dissipation of capacitors by electric biases, as shown in Figure 3. The bias applied to the system ranged from 20 V to 40 V using a power supply (TP-2303, TWINTEX Co., Ltd., Taipei, Taiwan). A pulsed bias was accomplished by switching to Relay-1 that was turned on in 0.5 s (capacitors charged) and turned off in 3 s (capacitors discharged). The capacitor discharged via Relay-2. The capacitor was overstressed via a series of charge and discharge to accumulate the degradation and propagate the heat generation in the interior of the capacitor. Relay-3 switched on to connect the capacitor to a function generator (GFG-3015, Good Will Instrument Co., Ltd., Taipei, Taiwan) that acted as an AC power supply with 5 Hz while Relay-2 switched off for further measuring capacitance from the amplitude change in the low pass filter. The relays were activated by the DAQ modulus of the NI LabVIEW system. A PZT cell, with dimensions of 3 mm square and a thickness of 200 μm, was used to act as an entropy and temperature sensor, and was attached to the top of the capsule capacitor by using polyimide tape for electrical insulation and silicone for installation, as shown in Figure 4. The current, voltage and temperature were acquired by using a precise measurement apparatus (Keysight N6705B) and an NI LabVIEW system (NI-PXIe 1082 and 8115). The measured data were further calculated to infer the entropy rate and variation.

## 3. Results and Discussion

Entropy generation plays an important role in electrochemical processes and irreversible thermodynamic processes, including heat engines, combustion and electrical devices with Joule dissipation. Entropy is an extensive property. It scales with volume and is immediately obtained from heat and temperature. The dimension of the entropy sensors is a critical issue to relate to the entropy generation. The thermal bath of silicon oil is a straightforward way to construct a thermodynamic model for further estimating the entropy generation rate (d*S*/d*t*) and entropy variation (Δ*S*). It is a reversible process, while the entropy released by the heat source of silicone oil (Δ*S_oil_*) is equal to the entropy absorbed by the PZT pyroelectric sensors (Δ*S_sensor_*). According to the second principle of thermodynamics, it is an irreversible process, while Δ*S_oil_* is not equal to Δ*S_sensor_* [18]. Therefore, Δ*S_sensor_* was obtained by the PZT cells with various dimensions. A PZT cell with an appropriate geometry was discussed to apply to the entropy sensors, and Δ*S_sensor_* could approach Δ*S_oil_* as much as possible under the easy probing of the induced electrical signals of the PZT cells. In short, a PZT cell with a smaller size is favorable to play an entropy sensor to avoid influencing the entropy variation of the measured objects, but it increases the difficulty in measuring the induced current due to diminution in the electrode area.

**Figure 5:**
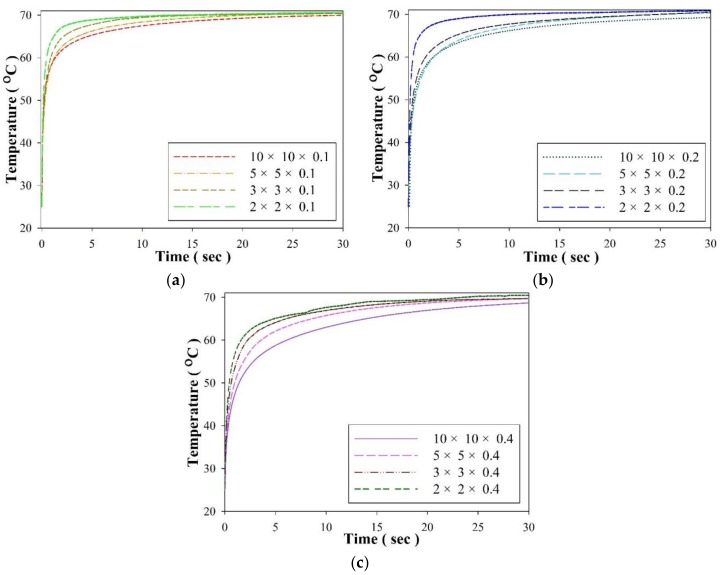
Temperature profiles of the PZT cells with various geometries and thicknesses of: (**a**) 0.1 mm, (**b**) 0.2 mm and (**c**) 0.4 mm in the thermal bath experiment.

**Figure 6:**
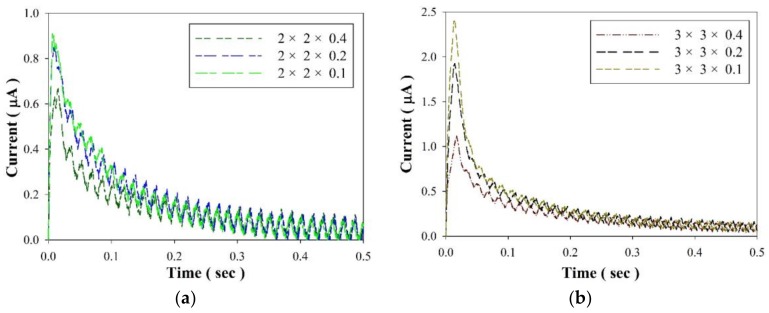

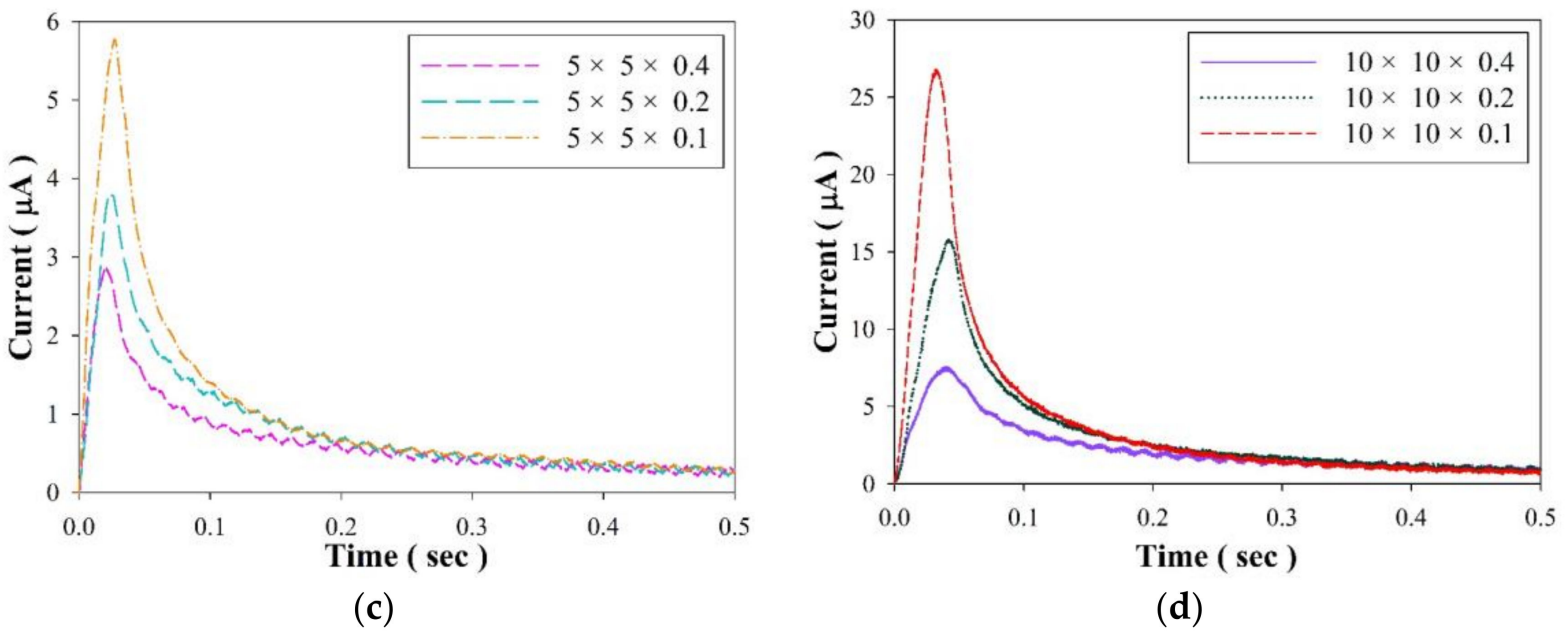
Induced currents of the PZT cells with various thicknesses and geometries of: (**a**) 2 mm × 2 mm, (**b**) 3 mm × 3 mm, (**c**) 5 mm × 5 mm and (**d**) 10 mm × 10 mm in the thermal bath experiment.

Figure 5 shows the temperature profile in the PZT cells with various geometries in the thermal bath experiments. It was evident that the temperature variation rate (d*T*/d*t*) was related to the volume of the PZT cells. The PZT cell with the larger volume had longer stability. Moreover, decreasing the thickness of the PZT cells was more effective than decreasing the surface area of the PZT cells for promoting the temperature variation rate. Hence, the PZT cell with the greater volume and thermal capacity was unsuitable to play the role of an entropy sensor because it would capture more thermal energies from the measured objects, further affecting the accuracy of the entropy variation and rate. Although the PZT cell with dimensions of 2 mm × 2 mm × 0.4 mm was able to promptly reach a stable temperature, it possessed a smaller electrode area and a lower temperature variation rate further reducing the induced current and electrical signals. Figure 6 shows the induced current of the PZT cells with various geometries in the experiment on thermal baths. The thinner PZT cell possessed a greater temperature variation rate to further promote the induced current while its electrode area was fixed. The electrode area of the PZT cells increased, and the induced current rose according to (1). Although increasing the electrode area was beneficial to enhance the induced current for reducing the difficulty of measurement, the PZT cell with the larger volume directly affected the entropy variation of the measured objects. The electrode area decreased to a 2 mm square; the induced current measurement was more difficult with the PZT cell due to background noises. Decreasing the thicknesses of the PZT cells had a positive effect on enhancing the induced current. The entropy variation was evaluated by the induced current of the pyroelectric cells. Hence, decreasing the electrode area of the PZT cells avoided affecting the entropy variation of the measured objects; the thickness of the PZT cells had to be further reduced to improve the temperature variation rate to strengthen the electrical signals.

**Figure 7:**
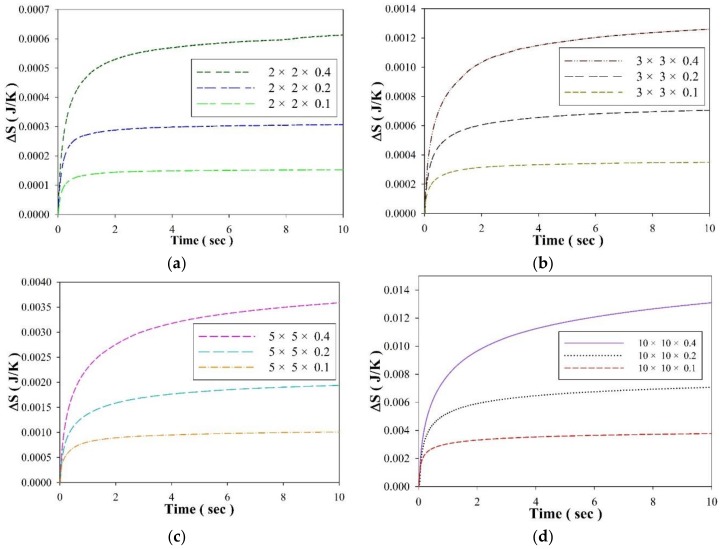
Entropy variations (ΔS) of the PZT cells with various thicknesses and geometries of: (**a**) 2 mm × 2 mm, (**b**) 3 mm × 3 mm, (**c**) 5 mm × 5 mm and (**d**) 10 mm × 10 mm in the thermal bath experiment.

Figure 7 shows the entropy variation of the PZT cells with various geometries in the thermal baths experiments. It was obvious that the entropy variation scaled with the volume of the PZT cells. A PZT cell 10 mm square generated an entropy variation of about 25 times that of a 2 mm square cell for the same PZT thickness of 100 μm. The entropy variation of a PZT cell with a larger volume had longer stability because it possessed a greater thermal capacity. Moreover, the PZT cell with dimensions of 2 mm × 2 mm × 0.1 mm possessed the smallest entropy variation because it had the least volume. In a word, a PZT cell with a smaller volume is advantageous for acting as an entropy sensor. Decreasing the electrode area of the PZT cell acted quadratically to reduce the volume of the cells. Nevertheless, the thickness of the PZT cell was related to the temperature variation rate. Reducing the thickness of the cell was useful in enhancing the temperature variation rate, and the entropy was estimated by the induced current of the pyroelectric cells. Hence, a PZT cell with a smaller electrode area was helpful for the design of entropy sensors, but its thickness had to be greatly reduced to promote the temperature variation rate and strengthen the electrical signals.

**Figure 8:**
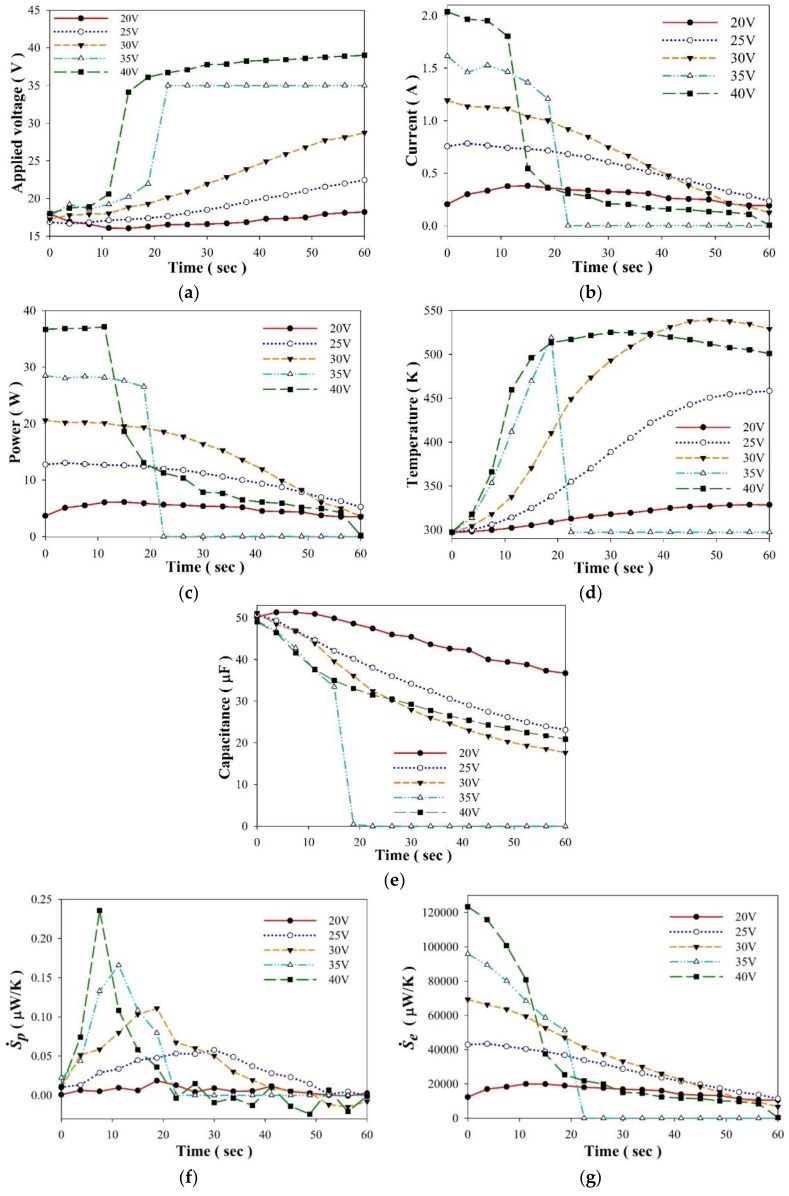
Time evolution of: (**a**) applied voltage, (**b**) current, (**c**) power, (**d**) temperature, (**e**) capacitance, (**f**) entropy generation rate (*Ś_p_*) by the pyroelectric cell, and (**g**) entropy generation rate (*Ś_e_*) by the electrical system for the selected capacitor with a capacitance of 47 μF and a maximum endured voltage of 4 V. The PZT cell had a dimension of 3 mm square and a thickness of 200 μm.

**Figure 9:**
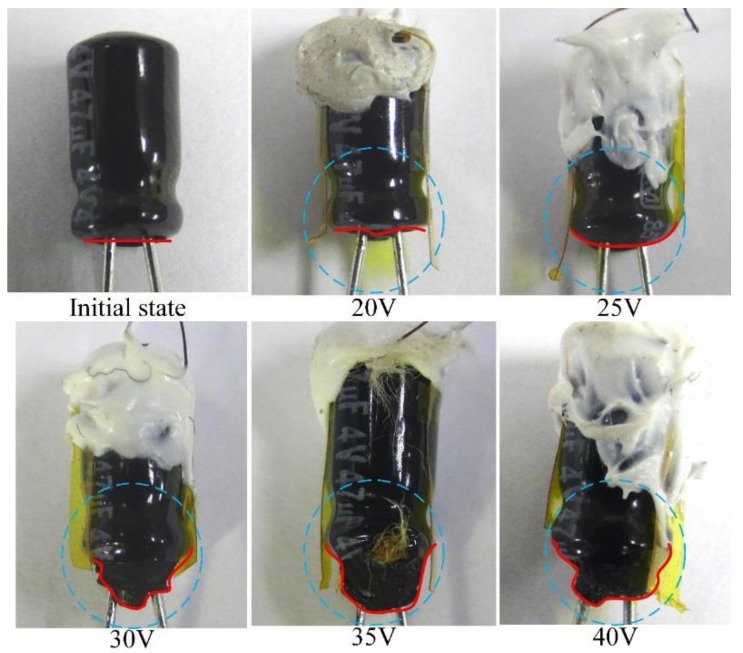
The final state of the capacitors with various applied voltages.

A PZT cell acting as an entropy sensor was further applied to appraise ageing in commercial electrolyte capacitors based on the measurement of the entropy generation rate. The time evolution of the current, voltage, power and capacitance for the selected capacitor were measured by the designed electrical system, shown in Figure 8. Furthermore, the entropy generation rate (*Ś_p_*) and temperature were estimated and measured by the PZT cell, and the entropy generation rate (*Ś_e_*) was calculated by the designed electrical system. Both *Ś_p_* and *Ś_e_* are depicted in Figure 8. The final capacitor state with various applied voltages is shown in Figure 9. The damage to the capacitor was more significant at higher applied voltages than at lower applied voltages. The power, current and temperature increased as the applied voltage increased. The capacitance was reduced when the applied voltage increased. Moreover, the rate of the rise in temperature was obviously proportional to the applied voltage. The input power increased, and then, the entropy generation rate increased. This meant that the capacitor was dissipating power. The damage state in the capacitor was closely related to the entropy generation rate. The capacitor obtained an open circuit when the current turned to zero, and the applied voltage was 35 V. Furthermore, the capacitance abruptly decreased at 17.5 s when the applied voltage was 35 V. This meant that the dielectric layer in the capacitor was destroyed in the applied voltage of 35 V. Although the drop in the time evolution of *Ś_e_* could implicate a time-to-failure of the capacitor, it was not apparent in the applied voltages of 25 V and 30 V. The threshold time was about 22.5 s for the applied voltages of 35 V and 40 V. The time evolution of *Ś_e_* always presented a tendency to decrease progressively due to the key experimental parameters of instantaneous power and temperature. However, the time evolution of *Ś_p_* always presented a mountain-like curve due to the induced current and temperature of the PZT cell. The threshold time for the drop time was also estimated by the time evolution of *Ś_p_* at about 22.5 s when the applied voltages were 35 V and 40 V. Nevertheless, the peak time in the time evolution of *Ś_p_* indeed presented the threshold time. The threshold time was not accurately predicted by the time evolution of *Ś_e_* in the applied voltages of 25 V and 30 V, but the threshold time could be easily estimated by the time evolution of *Ś_p_* at about 20 s and 30 s for the applied voltages of 30 V and 25 V, respectively. The threshold time was evaluated by the time evolution of *Ś_p_* at about 7.5 s and 11.25 s for the applied voltages of 40 V and 35 V, respectively. The peak value of *Ś_p_* increased as the applied voltage increased. Moreover, the peak value of *Ś_p_* could be used as the threshold entropy generation rate to demonstrate the failure mechanism. The peak values of *Ś_p_* were 0.235 μW/K, 0.165 μW/K, 0.11μW/K and 0.057 μW/K in the applied voltages of 40 V, 35 V, 30 V and 25 V, respectively. The value of *Ś_p_* was lower than that of *Ś_e_*. The peak value of *Ś_e_* was 0.51 × 10^6^ times than that of *Ś_p_* while the applied voltage was 40 V. This indicated that the PZT cell did not influence the entropy variation of the measured capacitor. Hence, *Ś_p_* was appraised by the PZT cell, and the pyroelectric cell was innately sensitive to the temperature variation rate, further generating the induced current. The pyroelectric cell is more suitable to act as an entropy sensor for predicting the aging and failure characterization of electrical or mechanical systems, and it could attribute to the peak value and time of *Ś_p_*.

## 4. Conclusions

An ageing mechanism can assist in predicting time-to-failure in systems, reactions, apparatuses or instruments. Moreover, the pyroelectric sensors materialize the estimation and monitoring of entropy generation in real time to further promote the performance of electrical or mechanical systems. The proposed entropy sensor can simultaneously probe temperature and heat according to the pyroelectric effect, so as to evaluate the entropy variation and rate of aging of systems. The entropy variation scaled with the volume of the PZT cells. A 10 mm square PZT cell generated an entropy variation about 25 times than that of a 2 mm square one for the same PZT thickness of 100 μm. Although a pyroelectric cell with a smaller electrode area was favorable to quadratically reduce its volume, its thickness had to be greatly reduced to increase the temperature variation rate and strengthen the induced current and electrical signals. The threshold time was evaluated by the time evolution of *Ś_p_* from the PZT cell at about 7.5 s, 11.25 s, 20 s and 30 s for the applied voltages of 40 V, 35 V, 30 V and 25 V respectively. The PZT cell did not influence the entropy variation of the measured capacitor. The pyroelectric cell could detect the peak value and time of the entropy generation rate, it was more suitable to act as an entropy sensor for predicting the aging and failure characterization of electrical or mechanical systems.

## Figures and Tables

**Table 1:** Properties of the bulk PZT sheet.

Item	Value
Pyroelectric coefficient (10^−4^∙C∙m^−2^∙K^−1^)	6.5
Relative dielectric constant (ε_33_^T/^ε_0_)	2100
Poling field (KV/cm)	35
Resistivity (Ohm-cm)	>10^12^
Density (g/cm^3^)	7.8
